# Photoionization Observables from Multi-Reference Dyson Orbitals Coupled to B-Spline DFT and TD-DFT Continuum

**DOI:** 10.3390/molecules27041203

**Published:** 2022-02-10

**Authors:** Bruno Nunes Cabral Tenorio, Aurora Ponzi, Sonia Coriani, Piero Decleva

**Affiliations:** 1 DTU Chemistry–Department of Chemistry, Technical University of Denmark, Kemitorvet Bldg 207, 2800 Kongens Lyngby, Denmark; 2 Department of Physical Chemistry, Ruđer Bošković Institute, 10000 Zagreb, Croatia; aurora.ponzi@irb.hr; 3 Istituto Officina dei Materiali IOM-CNR and Dipartimento di Scienze Chimiche e Farmaceutiche, Università degli Studi di Trieste, 34121 Trieste, Italy

**Keywords:** electron correlation, photoelectron spectroscopy, dyson orbitals, photoionization

## Abstract

We present a theoretical model to compute the accurate photoionization dynamical parameters (cross-sections, asymmetry parameters and orbital, or cross-section, ratios) from Dyson orbitals obtained with the multi-state complete active space perturbation theory to the second order (MS-CASPT2) method. Our new implementation of Dyson orbitals in OpenMolcas takes advantage of the full Abelian symmetry point group and has the corrected normalization. The Dyson orbitals are coupled to an accurate description of the electronic continuum obtained with a multicentric B-spline basis at the DFT and TD-DFT levels. Two prototype diatomic molecules, i.e., CS and SiS, have been chosen due to their smallness, which hides important correlation effects. These effects manifest themselves in the appearance of well-characterized isolated satellite bands in the middle of the valence region. The rich satellite structures make CS and SiS the perfect candidates for a computational study based on our highly accurate MS-CASPT2/B-spline TD-DFT protocol.

## 1. Introduction

The electronic structure problem is at the core of quantum chemistry, and the heart of most advanced simulation tools. Impressive advances have been brought over the years so that practical “black-box” tools are currently available to the general chemist. Despite the satisfactory performance of standard approaches, especially those based on density functional theory (DFT), in many applications the most accurate methods are based on correlated ab initio formalisms, the only ones that can address complex multiconfigurational situations. Notwithstanding the great advances in the treatment of the correlation problem, it remains the stumbling block of ab initio approaches and continues to be intensely researched. One focal point, discovered long ago [1,2,3], is the distinction between non-dynamical correlation, associated with quasi-degeneracies, and dynamical correlation, associated with the Coulomb cusp in the wavefunction when electrons coalesce. Being quite different in nature, they pose different demands on the approach, which are difficult to be met at the same time. Single reference approaches, like current coupled-cluster (CC) [4], are best at the dynamical problem, while multiconfigurational approaches [3] treat the quasidegeneracies well, but are less effective in describing the dynamical contribution. Moreover, even the adequate definition of the quasidegenerate space is often not trivial, and it requires an understanding of the basic features of the problem considered.

Electronic correlation is a theoretical concept associated with deviations from the independent particle picture. It is usually defined as the difference between the calculated results at the Hartree-Fock (HF) level and experiment, or between the HF results and the full configuration interaction (FCI) result, in the limit that the basis set approaches completeness. The single most important experimental technique for the study of the electronic structure that can reveal correlation effects in the ground and ionized states of atoms and molecules is photoelectron spectroscopy (PES). Photoelectron spectroscopy has given the most direct evidence of the soundness of the orbital structure of atoms and molecules, and, at the same time, ample evidence of correlation effects. One of the most direct pieces of evidence is the appearance of satellite states in the spectra, associated with multielectron excitations, i.e., bands forbidden at the Koopmans Theorem (KT) level [5,6]. In the core region, a prominent factor is electronic relaxation, i.e., the shrinking of the electron cloud in response to the formation of a core hole, although further correlation effects are still generally important. In the valence shell, relaxation is of minor importance, and correlation effects appear very neatly, the most prominent being associated to double excitations *one up-one down*, a deexcitation of an inner hole accompanied to an excitation into a virtual orbital, like, e.g., 3s${}^{-1}↔\;$3p${}^{-2}$3d, i.e., 3p${}^{2}\to$ 3s3d in Argon. It may be worth recalling that even relaxation can be considered a correlation effect, and not separately treated. Indeed, in several many-body schemes aiming at describing ionization, like Algebraic Diagrammatic Construction (ADC) [7] or equation-of-motion coupled cluster (EOM-CC) [8], relaxation is treated as pure correlation.

Unfortunately, a clear characterization of satellite states in the valence region is often difficult, as they emerge at relatively high energy, and are small-intensity features buried in a dense manifold of primary states. For this reason, although attracting great interest in the early times of photoelectron spectroscopy, their study has been sparsely pursued, both because of the difficulty of a clear experimental signature and the limitations of the theoretical tools available. In particular, dynamical studies of photoionization parameters, which convey a large amount of additional information beyond the bare energy position, are very few and have left questions still unanswered [9]. More recently, renewed interest has been devoted to very high energy satellites, relative to double core hole states, giving neat evidence of the presence of conjugate satellites, where the photon angular momentum is absorbed by the bound transition [10]. The most detailed information is ideally embodied in the molecular frame angular distributions (MFPADS) that probe in great detail the dynamic of the ionization. A comprehensive investigation has been conducted only in CO [11]. A recent study of the H${}_{2}$ molecule has shown the potential to image correlation contributions to the ground state wavefunction [12]. In most situations, photoionization is well described by a single channel final state, given by a coupled product of a bound wavefunction, describing the ionic state, and a continuum orbital for the photoelectron. In this model, the bound states are characterized by the Dyson and conjugate Dyson orbitals [13,14,15,16,17,18,19], and the photoionization process as transition from such orbital to the continuum. Thus, the dynamical observables are a detailed probe of such orbitals, which embody the change in correlation effects upon ionization. The calculation of Dyson orbitals has been implemented within several approaches. It is for instance available for EOM-CCSD in Q-Chem [20,21], in the $e^{T}$ program [22] for both EOM-CCSD and EOM-CC3 [23], for Green’s function in Gaussian [15], and in OpenMolcas [24,25,26,27] for CASSCF/RASSCF and CASPT2/RASPT2 wavefunctions.

Here, we compute photoionization observables by coupling CASPT2 Dyson orbitals to the molecular DFT and TD-DFT continuum obtained with our LCAO B-spline approach [28,29]. As prototype examples, we have chosen the CS and SiS molecules, due to their smallness which hides important correlation effects. These effects manifest themselves in the appearance of a well characterized isolated satellite band in the middle of the valence region, and even more well resolved satellites in SiS. Although these are not easy systems to measure, their photoelectron spectra have been obtained long ago [30,31], and they should be amenable to an experimental investigation of their photoionization properties with modern facilities. A previous study of CS has been presented [18], and another one for the CSe, CO, SiO, BF molecules [32], all isoelectronic in the valence shell with 10 electrons in three σ and one π orbitals, although all limited to the use of DFT continuum and CASSCF Dyson orbitals. Since for third row atoms the effect of TD-DFT on photoionization properties is quite substantial, we have chosen to reinvestigate it, as well as to test the improved calculation of Dyson orbitals. It is remarkable that the strongest effects among the diatomics considered are present in CS, but are anticipated to be even larger in SiS. This is a clear indication of variations in correlation effects even across a series of isoelectronic systems, and the significance of its understanding in terms of changes in their electronic structure.

## 2. Theory

### 2.1. Dyson Orbitals and Their Norms for a Biorthonormal Orbital Basis

The restricted active space state-interaction (RASSI) method has been used to compute matrix elements over distinct (biorthonormal) orbital sets [33,34].

Assume that we compute the initial and final states as two distinct wave functions $\Psi_{A}$ and $\Psi_{B}$, described by a CI expansion of Slater determinant (SD) functions $\Phi^{A}$ and $\Phi^{B}$ built with the sets of spin orbitals $\left\{\phi^{A}\right\}$ and $\left\{\phi^{B}\right\}$

(1) $$\Psi_{A}=\sum_{\mu }C_{\mu }^{A}\Phi_{\mu }^{A}\;\;,\;\;\;\;\Phi_{\mu }^{A}=a_{p}^{†}\cdots a_{s}^{†}|\mathrm{vac}\rangle$$

(2) $$\Psi_{B}=\sum_{\nu }C_{\nu }^{B}\Phi_{\nu }^{B}\;\;,\;\;\;\;\Phi_{\nu }^{B}=a_{q}^{†}\cdots a_{r}^{†}|\mathrm{vac}\rangle$$

where μ is a list of occupied spin orbitals (p⋯s) for a SD $\Phi_{\mu }$, and similarly for the index ν.

The overlap matrix between the wave functions $\Psi_{A}$ and $\Psi_{B}$ is given by

(3) $$\langle \Psi_{A}|\Psi_{B}\rangle =\sum_{\mu \nu }C_{\mu }^{A*}C_{\nu }^{B}S_{\mu \nu }$$

with $S_{\mu \nu }$ as the overlap matrix element between two SDs. This overlap matrix is not the unit matrix, since the spin orbitals of system A are not necessarily orthogonal to those of system B, $\langle \phi_{p}^{A}|\phi_{q}^{B}\rangle \neq \delta_{pq}$.

However, it is possible to find a set of non-unitary orbital transformations which transforms $\left\{\phi_{}^{A}\right\}$, $\left\{\phi_{}^{B}\right\}$, $\left\{C_{}^{A}\right\}$, $\left\{C_{}^{B}\right\}$ → $\left\{\tilde{\phi_{}^{A}}\right\}$, $\left\{\tilde{\phi_{}^{B}}\right\}$, $\left\{\tilde{C_{}^{A}}\right\}$, $\left\{\tilde{C_{}^{B}}\right\}$ such that $\langle \tilde{\phi_{p}^{A}}|\tilde{\phi_{q}^{B}}\rangle =\delta_{pq}$, where the overlap $S_{\mu \nu }$ becomes the unit matrix and the wave functions $\Psi_{A}$ and $\Psi_{B}$ remain the same. The non-unitary condition of the biorthonormal transformation also implies that the orbital overlaps $\langle \tilde{\phi_{p}^{A}}|\tilde{\phi_{q}^{A}}\rangle \neq \delta_{pq}$ and $\langle \tilde{\phi_{p}^{B}}|\tilde{\phi_{q}^{B}}\rangle \neq \delta_{pq}$, so we no longer have orthonormal sets.

Thus, using biorthonormally transformed orbital sets [33,34] allows us to apply simpler formulas when computing the overlap and matrix elements between different wave functions, that is,

(4) $$\langle \Psi_{A}|\Psi_{B}\rangle =\sum_{\mu }{\tilde{C}}_{\mu }^{A}{\tilde{C}}_{\mu }^{B}$$

which is the same expression as if a common orthonormal orbital set $\left\{\tilde{\phi }\right\}$ was used.

The Dyson orbital is a one-electron quantity intimately related to photoelectron spectroscopy and molecular ionization processes [15]. It is normally defined as overlap between the initial *N*-electron wavefunction, $\Psi_{I}^{N}$, and the final (N−1)-electron wave function, $\Psi_{F}^{N-1}$,

(5) $$\phi_{\mathrm{IF}}^{d}\left(x_{1}\right)=\sqrt{N}\int \Psi_{F}^{N-1}\left(x_{2},\cdots ,x_{N}\right)\Psi_{I}^{N}\left(x_{1},x_{2},\cdots ,x_{N}\right)dx_{2}\cdots dx_{N}$$

Using the state-interaction formalism [33,34], assuming that both the initial and the final states have been individually optimized, the Dyson orbital can be written as a linear combination of the biorthonormally-transformed orbital set (of the initial wavefunction)–that is,

(6) $$\phi_{\mathrm{IF}}^{d}=\sum_{q}\gamma_{q}^{\mathrm{IF}}{\tilde{\phi }}_{q}^{I}$$

where we defined the expansion coefficients as [13,14,19]

(7) $$\gamma_{q}^{\mathrm{IF}}=\langle \Psi_{F}^{N-1}|{\hat{a}}_{q}\Psi_{I}^{N}\rangle$$

(see, e.g., the SI of Ref. [19] for a derivation of the expression above.)

The coefficients $\gamma_{q}^{\mathrm{IF}}$ are easily computed using Equation (4) with $\Psi_{A}=\Psi_{F}^{N-1}$ and $\Psi_{B}={\hat{a}}_{q}\Psi_{I}^{N}$. One should note, however, that the annihilation operator ${\hat{a}}_{q}$ in Equation (7) is acting on the space of the transformed spin orbitals $\left\{{\tilde{\phi }}^{I}\right\}$ of $\Psi_{I}^{N}$. This implies that, albeit the Dyson coefficients $\gamma_{q}^{\mathrm{IF}}$ are conveniently obtained using biorthonormal orbitals through Equation (4), the squared norms of the Dyson orbitals, $|\phi_{\mathrm{IF}}^{d}{|}^{2}$, must take into consideration the non-orthonormality of the transformed $\left\{{\tilde{\phi }}^{I}\right\}$ set. This means that they cannot be computed simply as a sum of the squared expansion coefficients as otherwise typically done when using an orthonormal set. Instead, the squared norm of a Dyson orbital in a biorthonormally transformed orbital basis must be computed as

(8) $$|\phi_{\mathrm{IF}}^{d}{|}^{2}=\sum_{p,q}\gamma_{p}^{\mathrm{IF}*}\gamma_{q}^{\mathrm{IF}}\langle \tilde{\phi_{p}^{I}}|\tilde{\phi_{q}^{I}}\rangle$$

The Dyson orbitals, Equation (6), and their squared norms, Equation (8), have been implemented in a locally modified version of the OpenMolcas program package [25]. Different from the original implementation available in the OpenMolcas main repository [35], our Dyson orbital implementation corrects the norm for a biorthonormal set of molecular orbitals (Equation (8)), while taking advantage of full Abelian symmetry point group, which simplifies the calculations and orbital analysis.

### 2.2. The Continuum and the Calculation of the Photoionization Observables

The computational approach for the electronic continuum at the DFT and TD-DFT level, and the coupling to Dyson orbitals to obtain photoionization observables has been presented in detail in recent works [18,19,23,36], so we summarize here the most important points.

A special basis, employing B-splines as radial functions [37], and spherical harmonics for the angular part

(9) $$\chi_{ilm}=\frac{1}{r}B_{i}\left(r\right)Y_{lm}\left(\theta ,\phi \right)$$

allows one to obtain numerically accurate solutions of the Schrödinger equation also in the continuous spectrum. The basis comprises a long radial range, high angular momentum one-center expansion, and additional short-range expansions around the nuclei.

A DFT Kohn-Sham hamiltonian

(10) $$H_{\mathrm{KS}}=-\frac{1}{2}\Delta +V_{N}+V_{C}\left[\rho \right]+V_{\mathrm{XC}}\left[\rho \right]\;,$$

defined by the ground electron density ρ, is employed. In Equation (10), the first term represents the electron kinetic energy, $V_{N}$, $V_{C}$ and $V_{\mathrm{XC}}$ are, respectively, the nuclear attraction potential, the classical Coulomb potential and the DFT exchange correlation potential. The Kohn-Sham hamiltonian $H_{\mathrm{KS}}$ is represented by its matrix in the B-spline basis. The bound eigenvectors are obtained by a canonical diagonalization, while the full set of independent degenerate continuum eigenvectors are obtained, at each pre-selected energy, by a Galerkin approach [38,39]. Additionally, the linear response potential $V_{\lambda }^{\mathrm{SCF}}$ to the external dipole field $D_{\lambda }$ (Cartesian direction λ) is calculated at the TD-DFT level and employed in place of the bare external potential in the calculation of the dipole matrix elements

(11) $$D_{Elm\lambda }=\langle \phi_{Elm}|D_{\lambda }|\phi_{\mathrm{IF}}^{d}\rangle$$

where $\phi_{\mathrm{IF}}^{d}$ is the Dyson orbital relative to the initial and final states of interest previously defined [36]. With respect to DFT, TD-DFT includes channel coupling at the level of single-excitation configuration interaction, and some ground state correlation. These turn generally important for photoionization observables in the case of third row or heavier atoms, and at low kinetic energies. It is, however, computationally much more demanding than the pure single-particle static DFT approach, so that DFT is often the choice in the case of very large calculations. In any case, the comparison of the two approaches is useful to build experience on the accuracy and limitations of the pure DFT description.

Transforming from the angular momentum Elm representation to the linear momentum *k*, one obtains the dipole transition moment that allows the calculation of the differential photoionization cross section in the molecular frame

(12) $$\frac{d\sigma }{dk}=4\pi^{2}\alpha \omega {\left|D_{k\lambda }\right|}^{2}$$

from which standard angular momentum averaging finally gives the photoionization parameters σ and β of the well known formula

(13) $$\frac{d\sigma }{dk}=\frac{\sigma }{4\pi }\left(1+\beta P_{2}\left(\cos\theta \right)\right)$$

for randomly oriented molecules in the laboratory frame, with linearly polarized light and in the dipole approximation. Here θ is the angle between the electric vector and the photoelectron momentum, σ is the partial cross section and β is the asymmetry parameter.

## 3. Computational Details

Multi-state complete active space perturbation theory to second order (MS-CASPT2) [40,41,42,43] was used to compute the ionization energies and Dyson orbitals of the molecules CS and SiS. The neutral and ionized states were computed separately, whereas the Dyson orbitals were obtained within the state interaction formalism. Ground-state experimental equilibrium geometries, corresponding to bond lengths r${}_{eq}$ = 1.535 Å (CS) and 1.929 Å (SiS) were used [44]. The MS-CASPT2 calculations were carried out using the OpenMolcas package [25]. Ionization energies were also computed at the EOM-CCSD level using Q-Chem [20].

We used the aug-cc-pVTZ basis set [45,46] for all atoms. The active space selected for the CS molecule was formed by the valence orbitals 5–7σ and 1π, and including 3σ and 2π virtual orbitals. For the SiS molecule, we took the valence 7–9σ and the 3π orbital, complemented with the 3σ and 2π virtual orbitals to form the active space. Both active spaces used here for CS and SiS consist of 10 electrons and 12 orbitals. We will refer to this active space selection as CAS-10/12.

The molecular ground state densities were obtained from DFT calculations performed with the Amsterdam Density Functional (ADF) code [47] and the DZP basis set, using the LB94 exchange-correlation functional [48,49]. In both molecules, we employed a large one center basis with maximum expansion up to L${}_{max}=$ 20 with maximum range R${}_{max}=$ 25 a.u., and step size 0.125, which was supplemented by the smaller expansions centered on the atoms. The atomic expansion used on the CS molecule corresponds to L${}_{max}=$ 2 and R${}_{max}=$0.9 a.u. for both C and S atoms. For the SiS molecule, we used the atomic expansion L${}_{max}=2$ and R${}_{max}=0.8$ a.u. (Si) and R${}_{max}=1.2$ a.u. (S).

The Dyson orbitals obtained at the MS-CASPT2 level in a basis of Gaussian functions have been expanded in the B-spline basis by projection. Expanding the Dyson orbitals in a B-spline basis is convenient for easy evaluation of the one-electron matrix elements between bound and continuum orbitals.

## 4. Results

### 4.1. CS

The CS molecule has been the subject of recent theoretical investigations [18,23,50] due to its well known strong satellite structures [30]. A recent EOM-CC study [23] has shown that the EOM-CCSD approach is able to deal with ionizations dominated by one-hole (1h) configurations, but it fails to reproduce the satellites where two-holes/one-particle (2h1p) configurations are dominant, for which EOM-CC3 provided good results. As already anticipated, a previous study [18] of CS limited to the CASSCF level of treatment on the computation of the Dyson orbitals and DFT continuum has been presented. In that case [18] only the ionization energies (IEs) were corrected with the state-specific NEVPT2 method, although the Dyson orbitals—obtained at the CASSCF level—were missing important correlation description on the bound states, which can affect to some extent the photoionization properties. In the present study, both IEs and Dyson amplitudes are corrected with the MS-CASPT2 method and the continuum is further extended to TD-DFT, providing a better description of correlation in the photoionization dynamical parameters. An additional intense satellite is also investigated.

**Figure 1:**
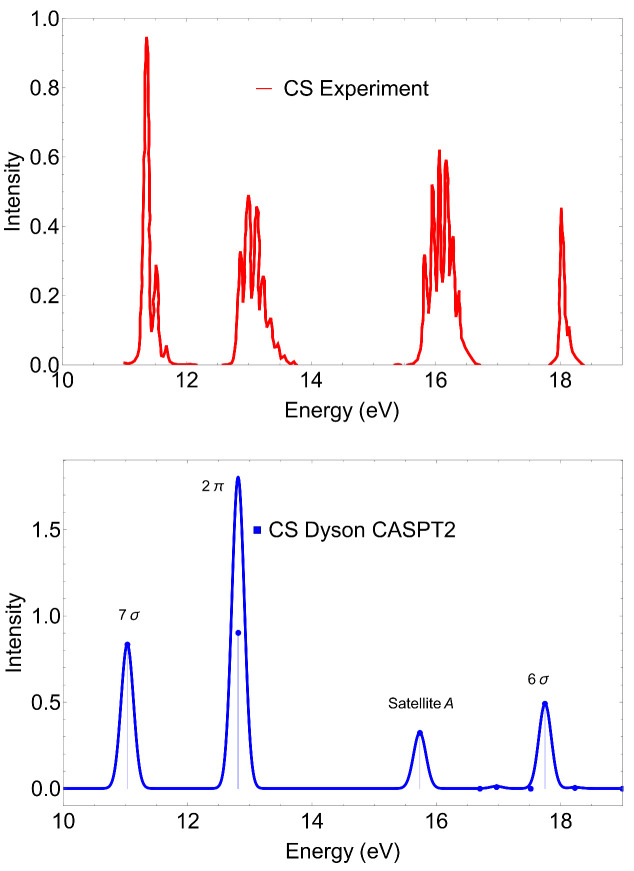
CS. PES obtained at the CASPT2 level of theory with the aug-cc-pVTZ basis set. The experimental PES was redigitized from Ref. [30].

The photoelectron spectrum of CS obtained at the MS-CASPT2 level of theory is shown in Figure 1 alongside with the experimental data [30]. In Table 1, we collect our calculated ionization energies, spectral strengths and state characterizations based on the weighted CI coefficients. Beside the MS-CASPT2 states, in Table 1 we also show the Koopmans theorem Hartree-Fock (HF) molecular orbital energies and EOM-CCSD ionization energies. The latter compilation further extends what was also reported in Ref. [23].

The first two peaks on the PES of CS, located at 11.33 and 12.79 eV, stem from the ionization of the HOMO (7σ) and HOMO-1 (2π) molecular orbitals, respectively. For these two peaks, the MS-CASPT2 and EOM-CCSD ionization energies and spectral strengths show reasonable agreement with the experiment and between each other, see Table 1. As these states are dominated by 1h configurations, EOM-CCSD is able to reproduce this type of ionization quite accurately, as it was also exemplified in Ref. [23]. For the satellite structures, where configurations of 2h1p type play an important (or the major) role, a better representation of double excitations (by perturbatively incorporating the effect of a higher excitation manifold) is required to obtain reasonable ionization energies, as Moitra et al. have recently demonstrated with use of the EOM-CC3 method [23]. Here, with the MS-CASPT2 level of approximation, we obtain IEs for the next two peaks within 0.3 eV of deviation relative to the experimental values. Notice that the deviations for the satellites IEs even for EOM-CC3 [23] were of the same magnitude or even higher. Nonetheless, the vibrational envelope must be taken into account if one seeks better correspondence with the experimental data. Since the goal of this study is primarily to demonstrate the capability of MS-CASPT2 Dyson orbitals coupled to the continuum representation provided by TD-DFT B-spline wave functions to yield photoionization dynamical observables, we ignore the coupling of vibrations with the photoionization.

According to MS-CASPT2, the two ionic states observed experimentally at 16 and 18 eV involve mainly the 1h ionization of the 6σ molecular orbital and the 2h1p configuration type 7$\sigma^{1}$2$\pi^{3}$3$\pi^{1}$. We observe that for the satellite state at 16 eV, the 2h1p configuration is dominant over the 1h configuration (EOM-CCSD locates it at 20.32 eV). For the second state, observed around 18 eV, the 1h and 2h1p configurations exchange roles and the 1h ionization becomes dominant. For this reason, the second state is often assigned as the primary peak arising from ionization of the 6σ molecular orbital [18].

An additional satellite not shown in the PES experiment is predicted by MS-CASPT2 at around 22.8 eV. This additional structure dominated by the 2h1p configuration type 6$\sigma^{1}$2$\pi^{3}$3$\pi^{1}$ is expected to be weak according to our calculated spectral strength, but very likely to be observed in an experiment. According to EOM-CCSD, satellite B is predicted at 24.83 eV and has a very strong 1*h* component from 5σ and larger spectral strength.

It is interesting to notice that although the ionization energies obtained with single-reference correlated methods like ADC(3) [50] and EOM-CC3 [23] compare quite well with our MS-CASPT2 and with previous CASSCF [18], the intensity ratio (also called pole strength ratio) between the pole strength of the first satellite (labeled here *Sat.A*) and the one of the main 6σ ionization, $R_{Sat.A}/R_{6\sigma }$, deviate substantially among these methods. To exemplify this, the intensity ratio was computed to be 0.26, 1.03, 0.49 and 0.66 at the ADC(3), EOM-CC3, CASSCF and MS-CASPT2 levels, respectively. This shows how sensitive these ratios are to the details of the correlation treatment, even in such a small system, how difficult it is to obtain accurate results, and the relevance of the study of satellite states to the development of correlated approaches. Actually, such ratios are not directly accessible experimentally, although they are reflected in the intensity of the ionization processes. In photoionization, the ratios involve a dipole matrix element to the continuum. In favourable situations, they can be reflected rather directly in the ratios of ionization cross sections [51]. Another technique closely connected is electron momentum spectroscopy [52].

**Figure 2:**
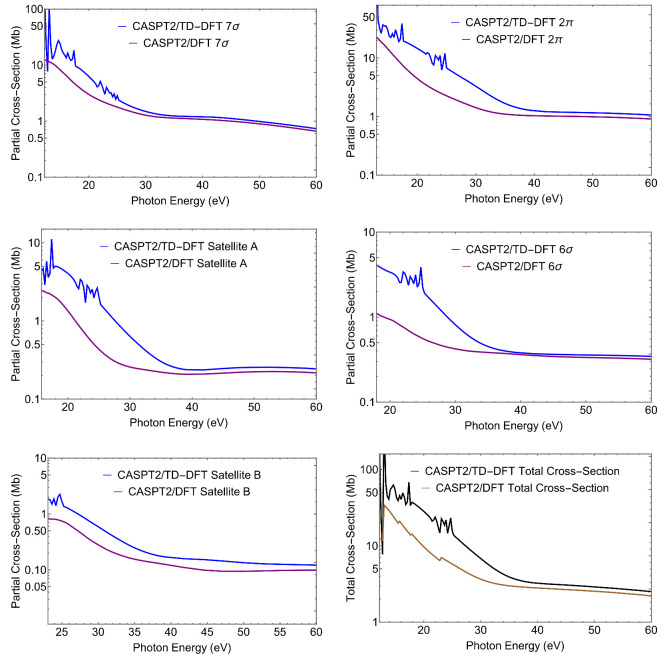
CS. CASPT2 Dyson/(TD-)DFT partial cross-sections (blue, TD-DFT and purple, DFT) relative to the ionic states considered. The last panel shows the total cross-section (black, TD-DFT and maroon, DFT).

**Figure 3:**
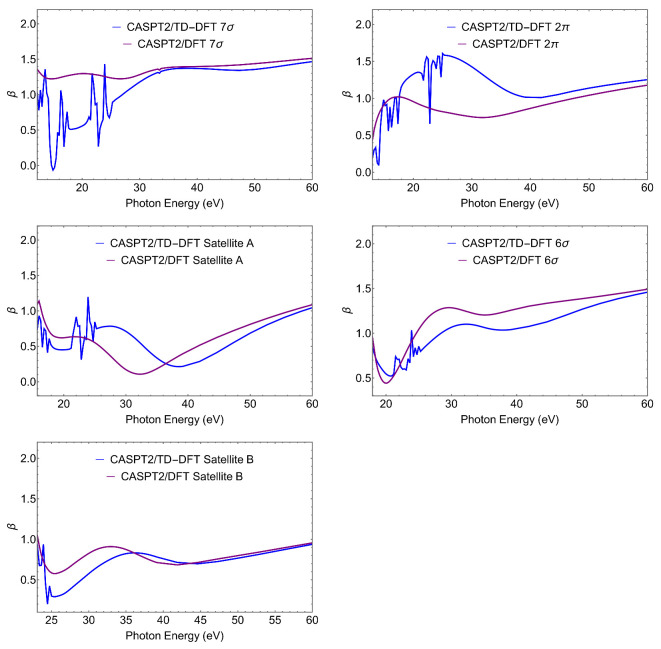
CS. CASPT2 Dyson/(TD-)DFT asymmetry parameters β relative to the ionic states considered.

Clearly, more information is contained in the full energy-dependent profiles of the photoionization observables. Here, we only present cross sections σ and asymmetry parameters β, that are the easiest to measure in a conventional photoionization experiment. Cross sections for the individual states and total cross section are shown in Figure 2. Asymmetry parameters are shown in Figure 3.

Let us first notice the large effect of TD-DFT at low energy both on σ and β, which is associated to the presence of a third row atom. TD-DFT cross sections are always higher than the DFT ones at low energies. This is a general behaviour, as interchannel coupling tends to redistribute intensity from higher to lower energy. Moreover TD-DFT includes oscillator strength associated with discrete excitations from lower lying orbitals that are embedded in the continuum, which appear as autoionization resonances with characteristic sharp peaks; they are absent in DFT. At higher energies, the effects are minor and DFT and TD-DFT become very close.

For what concerns the individual ionizations, one can note the much higher values at threshold for the outer valence ionizations 7σ and 2π, and the very different slopes of the cross sections. Also, the difference between DFT and TD-DFT is significantly less for the outer ionizations than for the next two states, but is again reduced for the second satellite. All these features reflect the different composition, *s* versus *p*, C versus S, atomic orbitals, of the respective Dyson orbitals. While it is hardly possible to extract their contribution from the photoionization profiles, the cross-comparison of the results obtained from different calculated Dyson orbitals as well as from experiment, is a sensitive probe of their nature (i.e., of the AO composition of the Dyson orbitals). The difference between the profiles of satellites A and B is quite striking, and reflects the calculated parentage, close to 6σ for the first and to 5σ for the second. Still also the profiles of the first satellite and the 6σ state are significantly different. All these behaviors are also apparent in the β parameter (Figure 3), which reflects interference among different partial waves in the continuum, and often highlights different aspects of the initial orbitals. We will not discuss this in detail, just notice again the rather different profiles of the first satellite and the 6σ ionization.

**Figure 4:**
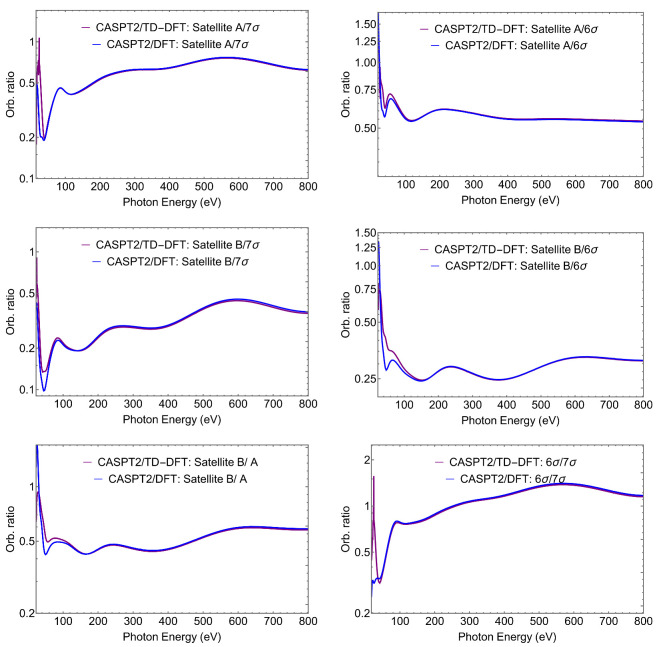
CS. CASPT2 Dyson/(TD-)DFT-continuum orbital ratios, $R_{IJ}=\sigma_{I}\left(\omega \right)/\sigma_{J}\left(\omega \right)$.

A simpler observable, which is also easier to measure experimentally, is the ratio between the cross sections relative to two ionic states *I* and *J*

$$R_{IJ}=\sigma_{I}\left(\omega \right)/\sigma_{J}\left(\omega \right)$$

also called ‘orbital ratio’. If the two ionic states, e.g., a main ionization and a satellite, shared the same Dyson orbital—as is often assumed when saying that the satellite “borrows intensity” from the former [6]—so that

$$\phi_{I}^{d}=C_{I}\varphi_{k}\;\;\;\;\;\;\phi_{J}^{d}=C_{J}\varphi_{k}$$

(where $\varphi_{k}$ is a generic orbital), then the two ionic states $\Psi_{I}$ and $\Psi_{J}$ would share the same profile, and in particular their orbital ratio

$$R_{IJ}=\frac{C_{I}}{C_{J}}=\frac{R_{I}}{R_{J}}$$

would be constant. So the (cross-section) ratios $R_{IJ}$ provide an immediate indicator of the parentage, or lack of it, of the two states considered. Some orbital ratios are presented in Figure 4, while a comparison with the intensity ratios computed at a few selected photon energies is presented in Table 2.

The orbital ratios are seen to vary considerably, and even at a rather high energy above threshold one can still notice conspicuous differences between the two values. The oscillations are particularly large at low energies, reflecting the difference in the wavefunctions of the two states, and the limitations of the borrowing model. The model is exact if a single determinant is used for the initial state, and each ionic state includes just a single 1h configuration. The Dyson orbital coincides then with the hole orbital, and its norm is the weight of the 1h configuration in the expansion. Correlation makes the picture more complex, although in many cases it is still a reasonable approximation. At low kinetic energies, the cross section is very sensitive even to small admixtures of other orbitals, and ratios vary considerably. At very large energies, however, it is the main AO components of the Dyson that dominate, and if these are similar for two ionic states, the ratio will stabilize to a final value. This can be observed in the ratio between Satellite A and the 6σ ionization. Both states share similar orbital characteristics, at least according to our assignments given in Table 1. The corresponding orbital ratio plotted in Figure 4 shows an approximately flat tail, which indeed suggests that Satellite A “borrows intensity” from the 6σ ionization by sharing similar orbital characteristics. Moreover, the orbital ratio at 800 eV approximates reasonably to the value of the intensity ratio $\left(R_{Sat.A}/R_{6\sigma }\right)$.

### 4.2. SiS

The photoelectron spectrum of SiS was obtained in Ref. [31], in high temperature experiments which also contain peaks from contaminating species. Theoretical studies are available in the literature of excited states of SiS, both neutral [53] and ionic [54], as well as a Green’s function (GF) study of the photoelectron spectrum [55]. Present results are reported in Table 3.

The first two ionic states are almost degenerate. Our CASPT2 calculations give a vertical I.E. for the $9\sigma^{}$ ionic state lower than the $3\pi^{}$ one; EOM-CCSD yields the same ordering, with a 0.12 eV separation between the two states. In Ref. [54], on the other hand, the adiabatic I.E. of the $3\pi^{}$ state was predicted to be lower than $9\sigma^{}$, with a separation of 0.15 eV. The same ordering was predicted in a GF study [55], although the separation was clearly overestimated by about 1 eV. The vibrational envelope of $3\pi^{}$ ionic state is quite extensive, and surrounds the sharper $9\sigma^{}$ one. The next band is observed at 13.88 eV in the experiment, and it is in good agreement with both our CASPT2 and EOM-CCSD results, as well as the GF ones (see also Ref. [54]). The next peak is calculated with CASPT2 at 15.30 eV (similar values from Refs. [54,55]), but is apparently missing in the experiment. However, a huge band attributed to an N${}_{2}$ contamination is present in the experimental spectrum at about this energy, which might have well obscured an SiS feature. At lower energies, two additional satellite bands were observed [31], the first in good agreement with the CASPT2 results, while the energy of the second appears overestimated by more than 1 eV. The situation becomes confused, with additional satellite states, all of sizable spectral strengths, appearing in the calculation. An analogous situation is reported in the rather old GF calculation, which appears to be only qualitative in this region. It signals a transition from outer valence $8\sigma^{}$ satellites to the breakdown of the orbital picture for the inner $7\sigma^{}$ ionization. Clearly SiS, with its two third-row atoms, has a richer satellite structure than CS. Moreover, from the CASPT2 results, it appears that the “main” $8\sigma^{}$ state is the third one, at 13.88 eV, which has the largest spectral strength, and the next is a satellite. The opposite is shown by the GF results. The former attribution may be rationalized by the Si atomic orbitals moving towards lower energies compared to carbon, so that the excited $9\sigma^{1}3\pi^{3}4\pi^{1}$ configuration is now higher in energy than the $8\sigma^{}$ one, leading to a reverse situation with respect to CS. In any case, the ionization intensity is more or less evenly split between the two states.

**Figure 5:**
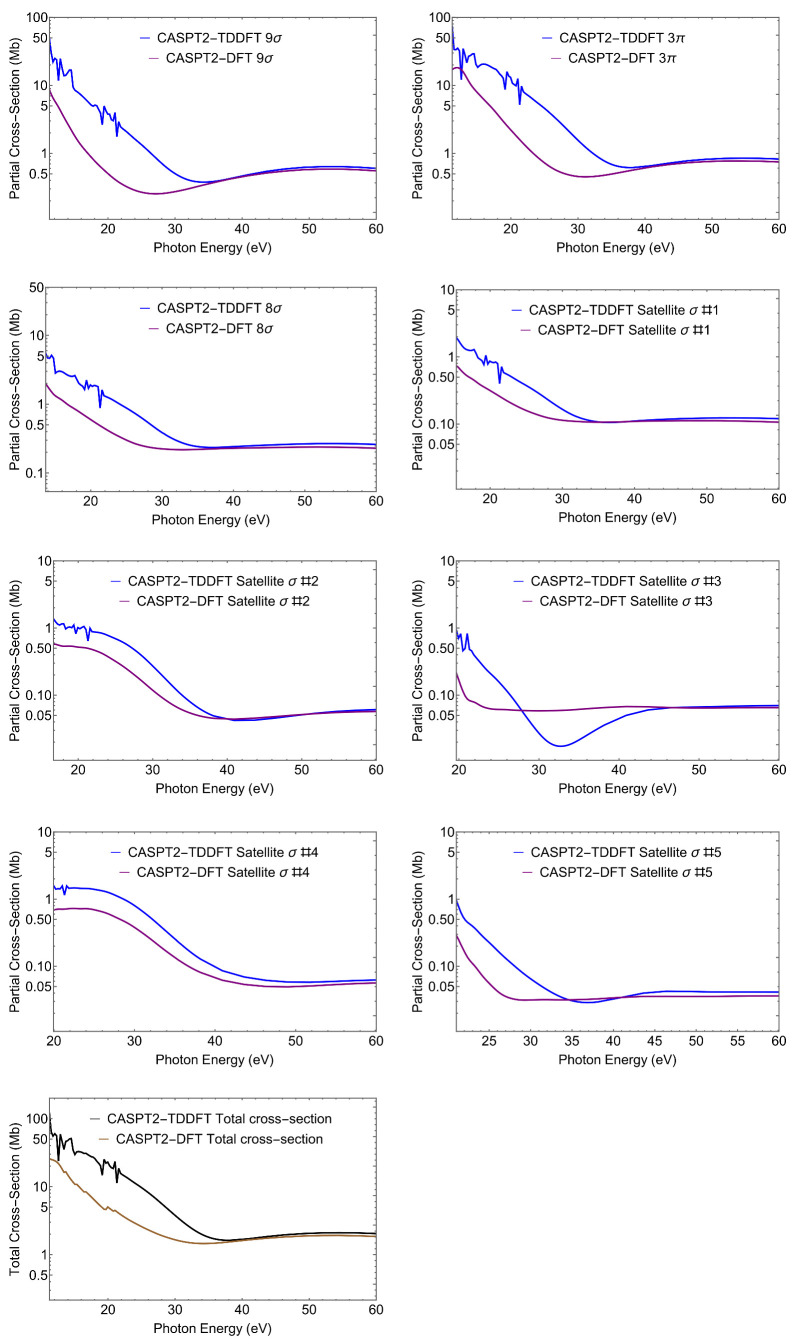
SiS. CASPT2 Dyson/(TD-)DFT cross-sections relative to the ionic states considered. The last panel shows the total cross-section (TD-DFT in black and DFT in maroon).

**Figure 6:**
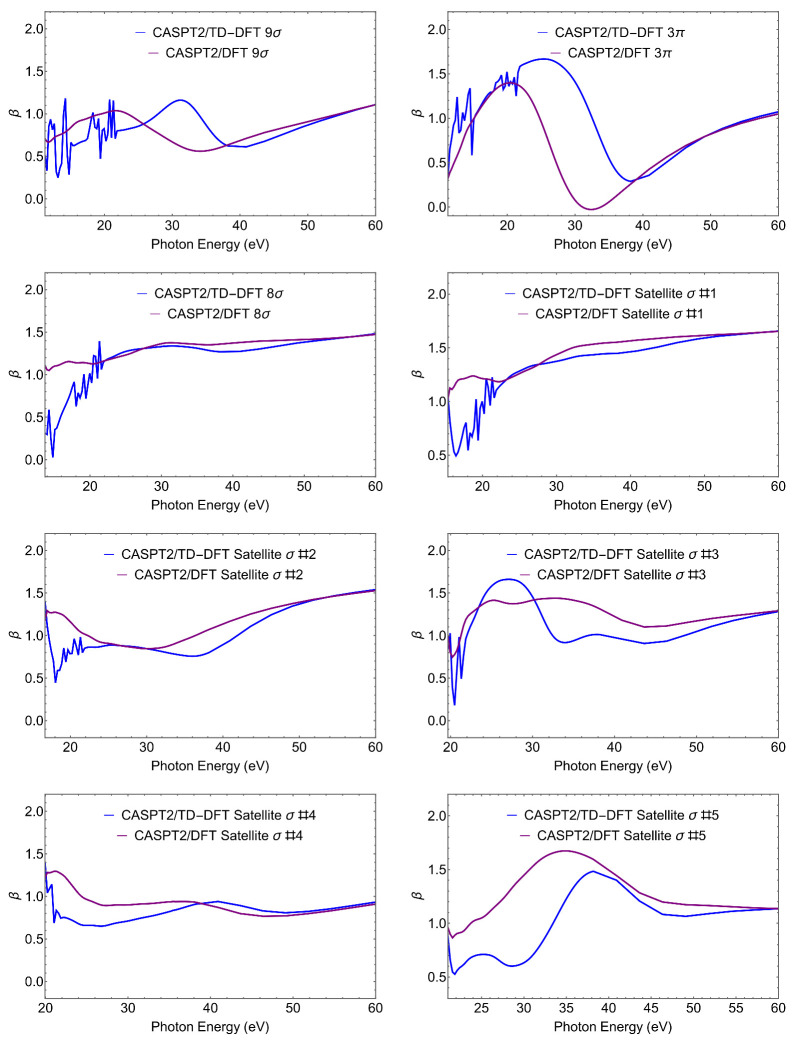
SiS. CASPT2 Dyson/(TD-)DFT asymmetry parameters β relative to the ionic states considered.

The cross sections, reported in Figure 5, look quite informative. The couple $8\sigma^{}$ and Satellite 1 show a quite similar profile, and so does the couple Satellite 2 and Satellite 4, although very different from that of the preceding one. A still distinct profile is shown by Satellite 3, while Satellite 5 seems somehow intermediate between the first group and the latter. It is noteworthy that the same behaviour is also shown by the β profiles, as reported in Figure 6. The β-profiles are more structured and the similarities less marked, yet they are still recognizable.

**Figure 7:**
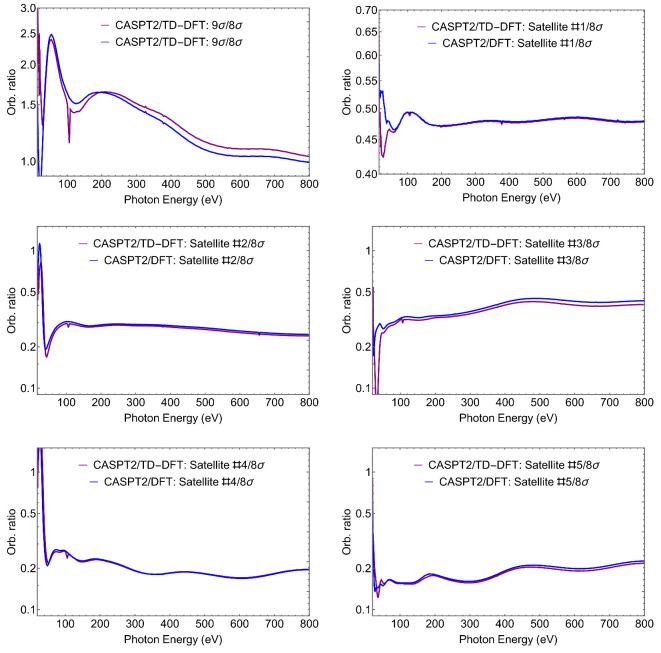
SiS. CASPT2-Dyson/(TD-)DFT-continuum orbital ratios, $R_{IJ}=\sigma_{I}\left(\omega \right)/\sigma_{J}\left(\omega \right)$.

To further the comparison, we have finally reported, in Figure 7, the cross-section ratios for all the satellites with respect to the third state, the $8\sigma^{}$ ionization. A comparison of the orbital ratios, at varying photon energies up to 800 eV, with the intensity ratios is presented in Table 4. The ratios between the satellites 1 to 5 and the 8σ at 800 eV coincide nicely with the intensity ratios reported in Table 4. From Figure 7, we also observe a flat tail on the satellite 1/8σ and satellite 2/8σ orbital ratios, which was expected as they show similar orbital characteristics according to our assignments on Table 3.

## 5. Conclusions

The calculation of the Dyson orbital from CASSCF/CASPT2 wavefunctions, taking into account full Abelian symmetry, has been implemented in OpenMolcas, and interfaced with a DFT/TD-DFT continuum on a B-spline basis, to allow for the calculation of photoionization cross sections with multiconfigurational bound wavefunctions. The approach yields an accurate description of photoionization in the case of highly correlated (and arguably also for complex open shell) bound states, both initial and final.

The methodology has been specifically applied to the description of satellite states in two small, but highly correlated, systems, CS and SiS. It is shown that describing the satellite states is a difficult correlation problem, and there is still a wide variance of results from current calculations already for the spectral strengths of the transitions. Photoionization profiles of the partial cross sections and asymmetry parameters, the simplest observables, are effective signatures of the corresponding Dyson orbitals, and constitute therefore an important test of the quality of the wavefunctions. Analysis of the cross section ratios between different channels highlights or disproves the similarity between “parent” and satellite wavefunctions and the “borrowing” mechanism. As a final note, with the same approach, other photoionization observables, e.g, the richer molecular frame angular distributions, MFPADS, can also be described. These, as well as the open-shell cases, will be the subject of future investigations.

## Figures and Tables

**Table 1:** CS. Ionisation energies I.E. (in eV) and pole strengths $R_{I}$ obtained at the MS-CASPT2 and EOM-CCSD levels with the aug-cc-pVTZ basis set. ${}^{†}$ The state characterization is based on the CI configurations with weight higher than 0.1. KT I.E. are the Koopmans theorem Hartree-Fock molecular orbital energies. Experimental values from Ref. [30].

Ionic State (*I*)	MS-CASPT2 State Character ${}^{†}$	Exp.	KT	MS-CASPT2		EOM-CCSD ^a^	
		I.E.	I.E.	I.E.	$R_{I}$	I.E.	$R_{I}$
7σ	7$\sigma^{1}$(0.79)	11.33	12.81	11.03	0.8354	11.52	0.8672
2π	2$\pi^{3}$(0.87)	12.79	12.58	12.81	0.9019	13.06	0.9091
Satellite A	7$\sigma^{1}$2$\pi^{3}$3$\pi^{1}$(0.50) + 6$\sigma^{1}$(0.28)	16.05		15.73	0.3242	20.32	0.1056
6σ	6$\sigma^{1}$(0.47) + 7$\sigma^{1}$2$\pi^{3}$3$\pi^{1}$(0.24)	18.00	18.85	17.75	0.4915	17.26	0.7683
Satellite B	5$\sigma^{1}$(0.17) + 6$\sigma^{1}$2$\pi^{3}$3$\pi^{1}$(0.22)			22.85	0.1810	24.83 ^b^	0.468

^a^ Some EOM-CCSD (and EOM-CC3) results can also be found in Ref. [23]. ^b^ Main composition according to EOM-CCSD: $5\sigma^{1}+7\sigma^{1}$ + 7$\sigma^{1}$2$\pi^{3}$3$\pi^{1}$ + 6$\sigma^{1}$2$\pi^{3}$3$\pi^{1}$.

**Table 2:** CS. Orbital ratio, $R_{IJ}=\sigma_{I}\left(\omega \right)/\sigma_{J}\left(\omega \right)$, at 30, 60, 400, 800 eV, and intensity ratio, $\frac{R_{I}}{R_{J}}$. The CASPT2-Dyson/TD-DFT-continuum approach was used for $R_{IJ}$, and CASPT2 for $R_{I}/R_{J}$.

Ionic States	$\frac{R_{I}}{R_{J}}$	Orbital Ratio ($R_{\mathrm{IJ}}$)			
*I/J*		30 eV	60 eV	400 eV	800 eV
6σ/7σ	0.59	0.50	0.47	1.16	1.10
Sat.A/7σ	0.39	0.30	0.35	0.64	0.56
Sat.B/7σ	0.21	0.43	0.16	0.29	0.32
Sat.A/6σ	0.66	0.76	0.70	0.55	0.55
Sat.B/6σ	0.37	0.71	0.35	0.25	0.32
Sat.B/Sat.A	0.55	0.91	0.50	0.45	0.57

**Table 3:** SiS. Ionisation energies (I.E., eV) and pole strengths $R_{I}$ obtained at the MS-CASPT2/aug-cc-pVTZ and EOM-CCSD/aug-cc-pVTZ levels of theory. ${}^{†}$ The state characterization is based on the CI configurations with weight higher than 0.1. KT I.E. are the Koopmans theorem Hartree-Fock molecular orbital energies. Experimental energies are from Ref. [31].

Ionic State (*I*)	MS-CASPT2 State Character ${}^{†}$	Exp.	KT	CASPT2		EOM-CCSD ^a^	
		I.E.	I.E.	I.E.	$R_{F}$	I.E.	$R_{I}$
9σ	9$\sigma^{1}$(0.74)	10.53	10.61	10.20	0.8882	10.49	0.8993 (9$\sigma^{1}$)
3π	3$\pi^{3}$(0.90)	10.56	10.57	10.50	0.8674	10.61	0.8939 (3$\pi^{3}$)
8σ	8$\sigma^{1}$(0.45) + 9$\sigma^{1}$3$\pi^{3}$4$\pi^{1}$(0.40)	13.88	15.65	13.54	0.4845	14.49	0.7776 (8$\sigma^{1}$)
Satellite #1	9$\sigma^{1}$3$\pi^{3}$4$\pi^{1}$(0.62) + 8$\sigma^{1}$(0.21)	-		15.30	0.2241		
Satellite #2	9$\sigma^{1}$3$\pi^{3}$4$\pi^{1}$(0.34) + 9$\sigma^{0}$10$\sigma^{1}$(0.14)	16.9		16.68	0.1122		
Satellite #3	7$\sigma^{1}$(0.16) + 9$\sigma^{1}$3$\pi^{2}$4$\pi^{2}$(0.22)	18.37		19.56	0.1597		
Satellite #4	9$\sigma^{0}$10$\sigma^{1}$(0.28)			19.88	0.1065		
Satellite #5	7$\sigma^{1}$(0.10) + 9$\sigma^{1}$3$\pi^{2}$4$\pi^{2}$(0.23)			20.92	0.0959		

^a^ Other I.E.’s with non-zero *R_I_* (in parenthesis): 17.51 eV (0.0005, Σ); 17.93 eV (0.0043, ∏); 18.03 eV (0.08054, Σ); 19.24 eV (0.00035, Σ); 20.58 eV (0.08913, Σ)—all of satellite character.

**Table 4:** SiS. Orbital ratio, $R_{IJ}=\sigma_{I}\left(\omega \right)/\sigma_{J}\left(\omega \right)$, at 30, 60, 400, 800 eV, and intensity ratio, $\frac{R_{I}}{R_{J}}$. The CASPT2-Dyson/TD-DFT-continuum approach was used for $R_{IJ}$, and CASPT2 for $R_{I}/R_{J}$.

Ionic States	$\frac{R_{I}}{R_{J}}$	Orbital Ratio ($R_{\mathrm{IJ}}$)			
*I/J*		30 eV	60 eV	400 eV	800 eV
9σ/8σ	1.83	1.13	2.4	1.2	1.04
Sat.1/8σ	0.46	0.43	0.46	0.47	0.48
Sat.2/8σ	0.23	0.68	0.24	0.28	0.24
Sat.3/8σ	0.33	0.09	0.27	0.40	0.41
Sat.4/8σ	0.22	1.48	0.24	0.18	0.20
Sat.5/8σ	0.20	0.14	0.15	0.19	0.22

## Data Availability

Data available from the authors upon request.

## Sample Availability

Not relevant.

## Abbreviations

The following abbreviations are used in this manuscript:

MS-CASPT2	Multi-State Complete Active Space Perturbation Theory to Second Order
CC	Coupled Cluster
EOM-CC	Equation of Motion Coupled Cluster
CCSD	Coupled Cluster Singles and Doubles
CC3	Coupled Cluster Singles, Doubles, and perturbative triples
ADC	Algebraic Diagrammatic Construction
DFT	Density Functional Theory
TD-DFT	Time-Dependent DFT
I.E.	Ionization Energy
PES	Photoelectron Spectroscopy
HF	Hartree-Fock
FCI	Full Configuration Interaction
MFPADS	Molecular Frame Photoelectron Angular Distributions
LCAO	Linear Combination of Atomic Orbitals
RASSI	Restricted Active Space State-Interaction
HOMO	Highest Occupied Molecular Orbital
KT	Koopmans’ Theorem
